# Accessibility of Primary, Specialist, and Allied Health Services for Aboriginal People Living in Rural and Remote Communities: Protocol for a Mixed-Methods Study

**DOI:** 10.2196/11471

**Published:** 2019-02-28

**Authors:** Rona Macniven, Kate Hunter, Michelle Lincoln, Ciaran O’Brien, Thomas Lee Jeffries Jr, Gregory Shein, Alexander Saxby, Donna Taylor, Tim Agius, Heather Finlayson, Robyn Martin, Kelvin Kong, Davida Nolan-Isles, Susannah Tobin, Kylie Gwynne

**Affiliations:** 1 Poche Centre for Indigenous Health Faculty of Medicine and Health The University of Sydney Sydney Australia; 2 The George Institute for Global Health The University of New South Wales Kensington Australia; 3 Faculty of Health University of Canberra Canberra Australia; 4 Prince of Wales Hospital Sydney Australia; 5 Royal Prince Alfred Hospital Sydney Australia; 6 Aboriginal Community Controlled Health Services Sydney Australia; 7 Western New South Wales Local Health District Dubbo Australia; 8 Mid North Coast Local Health District Port Macquarie Australia; 9 John Hunter Hospital New Lambton Heights Australia

**Keywords:** Aboriginal Australians, availability, accessibility, community-based health care services, health care services

## Abstract

**Background:**

Primary, specialist, and allied health services can assist in providing equitable access in rural and remote areas, where higher proportions of Aboriginal and Torres Strait Islander people (Aboriginal Australians) reside, to overcome the high rates of chronic diseases experienced by this population group. Little is currently known about the location and frequency of services and the extent to which providers believe delivery is occurring in a sustained and coordinated manner.

**Objective:**

The objective of this study will be to determine the availability, accessibility, and level of coordination of a range of community-based health care services to Aboriginal people and identify potential barriers in accessing health care services from the perspectives of the health service providers.

**Methods:**

This mixed-methods study will take place in 3 deidentified communities in New South Wales selected for their high population of Aboriginal people and geographical representation of location type (coastal, rural, and border). The study is designed and will be conducted in collaboration with the communities, Aboriginal Community Controlled Health Services (ACCHSs), and other local health services. Data collection will involve face-to-face and telephone interviews with participants who are health and community professionals and stakeholders. Participants will be recruited through snowball sampling and will answer structured, quantitative questions about the availability and accessibility of primary health care, specialist medical and allied health services and qualitative questions about accessing services. Quantitative data analysis will determine the frequency and accessibility of specific services across each community. Thematic and content analysis will identify issues relating to availability, accessibility, and coordination arising from the qualitative data. We will then combine the quantitative and qualitative data using a health ecosystems approach.

**Results:**

We identified 28 stakeholder participants across the ACCHSs for recruitment through snowball sampling (coastal, n=4; rural, n=12; and border, n=12) for data collection. The project was funded in 2017, and enrolment was completed in 2017. Data analysis is currently under way, and the first results are expected to be submitted for publication in 2019.

**Conclusions:**

The study will give an indication of the scope and level of coordination of primary, specialist, and allied health services in rural communities with high Aboriginal populations from the perspectives of service providers from those communities. Identification of factors affecting the availability, accessibility, and coordination of services can assist ways of developing and implementing culturally sensitive service delivery. These findings could inform recommendations for the provision of health services for Aboriginal people in rural and remote settings. The study will also contribute to the broader literature of rural and remote health service provision.

**International Registered Report Identifier (IRRID):**

DERR1-10.2196/11471

## Introduction

Access to coordinated and timely specialist care improves health outcomes for people with complex health needs [1]. During 2010-2012, the estimated life expectancy at birth for Aboriginal and Torres Strait Islander Australians (hereafter Aboriginal Australians) was 10 years lower than that for non-Aboriginal Australians [2]. Much of this gap in life expectancy between Aboriginal Australians and non-Aboriginal Australians has been attributed to chronic diseases [3]. The rates of these chronic diseases are considerably higher among Aboriginal people than among the overall Australian population [4]. In 2008, a 25-year political commitment called Closing the Gap was made through the establishment of seven targets across health, education, and employment, yet only modest progress has been achieved over almost a decade [5].

Approximately one-third of the Australian population lives outside the eight major cities, roughly 7.7 million people [6]. However, a significant disparity between the decentralization of the population and health expenditure exists both between urban versus rural and regional and remote areas [7] as well as among rural or regional and remote comparative areas. Nationally, age-standardized services for very remote areas were funded at less than a third of the amount received by major cities [8]. This may lead to inequity in both the funding and provision of health services in these regions. The state of New South Wales (NSW) is home to the largest proportion of Australia’s Aboriginal population, 65% of which live outside metropolitan areas compared with only 25% of the non-Aboriginal population [9]. Large tertiary medical centers located in metropolitan areas can be accessed by the entire population, yet time and cost barriers in traveling long distances to facilities can be prohibitive, particularly for socioeconomically disadvantaged groups such as Aboriginal Australians.

Tackling chronic diseases requires a multidisciplinary approach [10], with regular, appropriate consultation, treatment, and support. Various policy initiatives have been developed at the national, state, and local level to respond to this situation. At the national level, the mainstream strategy outlines the need to manage chronic diseases among Aboriginal Australians and indicates that access to appropriate services is vital for reducing the burden of disease [11]. Providing such services will not only reduce the burden of chronic diseases to communities and the country at large but also impact the quality and longevity of life for Aboriginal Australians significantly [5]. The implementation of the most recent, long-term national Aboriginal health plan focuses on improving the health system through more comprehensive, culturally competent, and effective services, including investing in increased capability of Aboriginal Community Controlled Health Services (ACCHSs) to meet identified needs [12]. Ensuring that services are adequately resourced is vital to ensuring this outcome is achieved [13,14].

The unique social and cultural needs of Aboriginal Australians should be carefully considered in the provision of care to ensure culturally competent service delivery [15]. The National Aboriginal Community Controlled Health Organization, the Aboriginal Health and Medical Research Council (AHMRC), and rural ACCHSs have led to significant improvements in the delivery of health care to Aboriginal Australians [16,17]. However, the capacity of ACCHSs are limited, and most health care services are provided to Aboriginal people through mainstream health care services [15]. The coordination of services across multiple agencies and health problems is challenging. An ecosystems approach provides a way to think about health issues as a whole through a system lens, enabling integrated responses across and between health services and health issues. A health ecosystem approach brings key stakeholders together to form partnerships and facilitates engagement between all relevant sectors necessary for connected health care to occur [18,19]. The approach has been used to help conceptualize and understand health in its wider environmental or ecosystem context and provide innovation in health care [19,20]. Determining the location and frequency of current health care services over a geographical jurisdiction allows for the identification of targeted areas for coordinated future service provision to create a fairer spread of care across the population.

The primary aim of this study is to determine the availability and accessibility of primary health care, specialist medical, and allied health services to Aboriginal people living in three rural or remote towns in NSW and their current level of coordination from the perspectives of service providers in those communities.

## Methods

### Study Design

#### Study Approach

Our study design will utilize a mixed-methods approach to give a broader, more comprehensive perspective to answer the aims of the study and combine the strengths of quantitative and qualitative approaches. We will use both inductive and deductive approaches, using initial deductive reasoning driven by the examination of the aims of the study in combination with generating findings that emerge from observations from the data. The study will take place in 3 communities (coastal, remote, and border) across NSW with higher than average populations of Aboriginal people [9]. These three communities have been selected based on their locations being representative of centers with varying access to metropolitan health services and each having a strong history of community-driven health service development. The investigators have existing, established relationships with the three communities, built up over several years of collaborative working and co-designed research. The coastal, remote, and border communities have populations of 14,000 (17% Aboriginal), 1400 (65% Aboriginal), and 500 (63% Aboriginal) people, respectively [6,9].

We will conduct the study in collaboration with ACCHSs and other service providers including hospitals, general practice clinics, pharmacies, and community health centers. Stakeholder participants will be staff recruited from local health districts and primary health networks, ACCHSs, pharmacies, early childhood centers, general practices, Aboriginal working parties, schools, local councils, and the private health sector. By interviewing representatives from multiple service providers in each community, we will be able to triangulate the data to ensure that we capture a comprehensive picture of service providers’ perspectives.

#### Phase 1: Community Engagement and Recruitment

**Figure 1:**
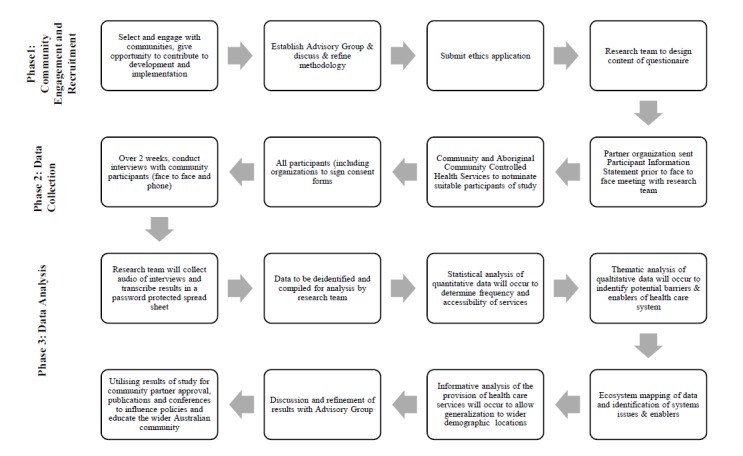
Flow chart of the study.

Figure 1 presents a flowchart of the study phases. In Phase 1, the researchers approached ACCHS stakeholders in the three selected locations to participate in the study. We gave these stakeholders the opportunity to join the research team and contribute to the development and implementation of the research design and data collection tools. We have established an independent study advisory group to oversee the study and its governance [21]. The advisory group comprises both Aboriginal and non-Aboriginal professionals and community members who have knowledge and expertise relating to at least one of the three communities. This group can enhance the reflexivity of the research process by guiding the researchers during each of the three phases. Each organization involved in the study was asked to nominate individual participants who meet the study inclusion criteria of having existing relationships within the communities and knowledge of and access to information regarding the provision of services to these communities.

While we selected the three communities for the study based on their demographic characteristics and their established relationships with the researchers, a snowball sampling recruitment process will be used to recruit individual participants. We asked partner ACCHSs to provide information on all relevant service providers in their community for recruitment. Subsequently, the research team contacted each of these organizations to invite them to participate in the study. Where consent was granted by the organization to approach their staff members to participate in the study, the organization subsequently introduced the study to their staff and facilitated recruitment by providing contact details of potential participants. While snowball sampling does not provide a known sampling population size, it gives a more effective means of accessing vulnerable and isolated population groups, such as rural populations and professionals (including those who identify as Aboriginal) working with Aboriginal communities, for mixed-methods research [22].

#### Phase 2: Data Collection

We will deidentify each location to ensure confidentiality and privacy for the participants, which may facilitate more honest and candid responses, and to protect the privacy of the communities. All participants will go through an informed consent process and sign consent forms prior to data collection. We will collect the data over a 2-week period in each community using both face-to-face and telephone interviews. Each community supported the undertaking of data collection in this timeframe and considered it to be appropriate for answering the aims of the study and that it would not place an undue burden on the community. We will invite the participants to undertake a structured, quantitative interview about the primary health care, specialist medical and allied health services in their community and give them the opportunity to provide longer, open-ended (qualitative) answers for each question. The quantitative component will allow the participants to indicate the specific services (primary, specialist, and allied health services, eg, podiatry, renal nurse, or sonography) available in these communities, by providing a response of “yes,” “no,” or “unsure.” Participants will be asked to identify where (through giving an open-ended response, eg, ACCHS or local hospital) these services are offered. We will also ask the participants about the frequency of service delivery, measured through a 9-point scale from “always” to “never” (including the responses “weekly,” “monthly,” and “annually”). The qualitative component will collect information about the participant’s experiences, beliefs, and expectations about accessing health services in their community. Participants will also be asked open-ended questions about the barriers and enablers to health care services for Aboriginal people in their respective locations.

One of the authors and a project manager, who are experienced in conducting interviews for qualitative research in Aboriginal communities, will conduct the interviews. All interviews will be audio recorded and transcribed by a transcription service. Quantitative data will be collected using REDCap (REDCap Software), a secure electronic data capture tool hosted at The George Institute for Global Health [23].

#### Phase 3: Data Analysis

We will analyze the data to provide an informative analysis of service providers’ perceptions of the provision of health care services to these communities. A research student who was not involved in the data collection will conduct the initial data analyses, with subsequent data interpretation by the study investigators and the advisory group.

Descriptive statistical analysis will be used for all quantitative data to determine the frequency of specific services, across the three communities and combined, to determine their availability. We anticipate that data on services will be defined and categorized by service type (eg, primary, specialist, or allied), service delivery type (eg, nurse, podiatrist, or sonographer), and delivery organization (eg, public or private). In each community, we will compare participant reports of the type and frequency of health services available. We will tabulate services readily identified by all participants as well as those where there is uncertainty or dispute. Similarly, the reported frequency of services will be compared across participants in each community to determine knowledge about services available at a community level.

All qualitative data will undergo thematic and content analysis using both inductive and deductive approaches [24]. This analysis will include the identification of any potential barriers or enablers or broader emerging themes relating to the provision of and access to health services for Aboriginal people. The qualitative and quantitative data will be visually combined and mapped using a health ecosystem approach [18]. This will provide an ecological framework [25] to analyze how the various parts of the health care systems in this study, such as perceptions of coordination, funding, and design, interact. A member-checking [26] process will subsequently be undertaken through consultation with the advisory group to examine the validity of the findings and ensure reflexivity has occurred across the study.

Quantitative analyses will be performed using SPSS 19.0 (IBM Corp) and qualitative analyses using NVivo 10 (QSR International).

### Ethics and Governance

The AHMRC of NSW gave ethical approval for this study (1173/16). A requirement of ethical approval was the establishment of an advisory group to advise the research team. The advisory committee comprised representatives from ACCHSs and mainstream (including rural-specific) health organizations. This committee met twice at the development and start-up of the study and will meet again once data collection is complete. The study addresses the AHMRC and National Health and Medical Research Council principles and guidelines for Aboriginal and Torres Strait Islander research [21]. We will share the findings of this study with the three Aboriginal communities and their ACCHSs, the advisory group supporting the study, and the AHMRC as well as in peer-reviewed publications and at conferences.

## Results

We identified 28 stakeholder participants across the ACCHSs for recruitment through snowball sampling (n=4 coastal, n=12 rural, and n=12 border) in Phase 1 for data collection in Phase 2. The project was funded in 2017, and enrolment was completed in 2017. Data analysis is currently underway, and the first results are expected to be submitted for publication in 2019.

## Discussion

This study will use quantitative and qualitative data to provide unique insight into the lived experiences of service providers in 3 rural communities with high Aboriginal populations in NSW. Further, this study provides insights into the availability and frequency of primary, specialist, and allied health services in those communities. Combining the quantitative and qualitative data will provide a comprehensive way to identify issues and enablers from a systems perspective and make explicit how all the parts of the health system might interact, support, and influence outcomes [25]. The results of this study may identify barriers and enablers of health services in rural and remote communities. This information will help inform recommendations about how to improve health care services to Aboriginal people. Subsequently, this will contribute to easing the burden of chronic diseases for people in these communities specifically and to other nonmetropolitan jurisdictions across and beyond Australia.

The provision of health care services to Australians is a multifaceted undertaking that is often influenced by a range of factors, particularly social, cultural, political, and geographical factors. When this provision exists outside major cities, the challenges and expenses faced by health care providers are significantly increased [8]. The provision of coordinated primary, specialist, and allied health services may help overcome some of the barriers relating to access to services and in turn improve health outcomes among Aboriginal people living in rural and remote areas [27]. These barriers include geographic remoteness, socioeconomic factors, cultural competence of services, and specialist availability [28] in addition to the general limitations of public and primary health care systems [29], such as waiting times and hospital service structure.

The strengths of this study are its collaborative development and delivery, with the key stakeholders involved in health service delivery to Aboriginal Australians including community-controlled health services. Another strength is the application of the novel ecosystems approach that enables a broader lens on the interactions between individuals, issues, and system [19,20]. Limitations may include bias relating to the representativeness of the participant sample and the validity of self-report responses as well as the interpretation of qualitative data by the researchers. We will attempt to ameliorate any biases through member-checking of the results and undertaking snowball sampling until data saturation is reached. The study is the first of its kind to comprehensively map the primary, specialist and allied health activities in identified geographical areas of need for a high priority population group from the perspectives of service providers. Ultimately, this study will provide insights into how to better provide health care services for Aboriginal people in rural and remote communities. The findings will also contribute to the broader literature of rural and remote health service provision [30] and provide recommendations for future practice, which if adopted could lead to improvement of population health services.

## Abbreviations

ACCHS: Aboriginal Community Controlled Health Service
AHMRC: Aboriginal Health and Medical Research Council
NSW: New South Wales
